# Actin cytoskeleton assembly regulates collagen production via TGF‐β type II receptor in human skin fibroblasts

**DOI:** 10.1111/jcmm.13685

**Published:** 2018-06-11

**Authors:** Zhaoping Qin, Gary J. Fisher, John J. Voorhees, Taihao Quan

**Affiliations:** ^1^ Department of Dermatology University of Michigan Medical School Ann Arbor MI USA

**Keywords:** collagen, cytoskeleton, extracellular matrix, skin ageing, TGF‐β signalling

## Abstract

The dermal compartment of skin is primarily composed of collagen‐rich extracellular matrix (ECM), which is produced by dermal fibroblasts. In Young skin, fibroblasts attach to the ECM through integrins. During ageing, fragmentation of the dermal ECM limits fibroblast attachment. This reduced attachment is associated with decreased collagen production, a major cause of skin thinning and fragility, in the elderly. Fibroblast attachment promotes assembly of the cellular actin cytoskeleton, which generates mechanical forces needed for structural support. The mechanism(s) linking reduced assembly of the actin cytoskeleton to decreased collagen production remains unclear. Here, we report that disassembly of the actin cytoskeleton results in impairment of TGF‐β pathway, which controls collagen production, in dermal fibroblasts. Cytoskeleton disassembly rapidly down‐regulates TGF‐β type II receptor (TβRII) levels. This down‐regulation leads to reduced activation of downstream effectors Smad2/Smad3 and CCN2, resulting in decreased collagen production. These responses are fully reversible; restoration of actin cytoskeleton assembly up‐regulates TβRII, Smad2/Smad3, CCN2 and collagen expression. Finally, actin cytoskeleton‐dependent reduction of TβRII is mediated by induction of microRNA 21, a potent inhibitor of TβRII protein expression. Our findings reveal a novel mechanism that links actin cytoskeleton assembly and collagen expression in dermal fibroblasts. This mechanism likely contributes to loss of TβRII and collagen production, which are observed in aged human skin.

## 1 INTRODUCTION

Loss of tissue mass and accumulation of tissue damage are common features of ageing of many vital tissues.^1^, ^2^ In human skin, these age‐associated features are readily observable as thinning and fragility. The dermal extracellular matrix (ECM), which is composed primarily of type I collagen fibrils, provides the majority of skin mass, and is responsible for structural/mechanical support.^3^ Increased fragmentation and reduced production of type I collagen fibrils are prominent features of the skin dermis in aged individuals.^4^, ^5^, ^6^, ^7^, ^8^, ^9^ Age‐associated alterations of collagen fibrils also create a tissue microenvironment that compromises skin health by impairing vasculature function,^2^, ^10^, ^11^ delaying wound healing^12^, ^13^, ^14^ and promoting skin cancer.^15^, ^16^

In skin, fibroblasts are primarily responsible for synthesis of the dermal ECM. In young healthy skin, dermal fibroblasts attach to intact, dense collagen fibrils, through integrin collagen receptors. This attachment promotes assembly of the cellular actin cytoskeleton, which generates mechanical forces that give rise to fibroblast morphology.^6^, ^17^, ^18^, ^19^, ^20^ During ageing, collagen fibrils become fragmented, less dense and disorganized.^17^ Fragmentation destroys attachment sites, thereby diminishing the assembly of the actin cytoskeleton within fibroblasts.

Assembly of the actin cytoskeleton, following integrin‐dependent attachment of fibroblasts to the dermal ECM, is a highly complex process involving the co‐ordinated assembly of a large number of cytoskeletal proteins.^21^, ^22^, ^23^, ^24^, ^25^ Central to this process is the dynamic, reversible polymerization of actin monomers to form microfilaments that provide critical intracellular mechanical support.^26^, ^27^, ^28^

Production of ECM proteins by dermal fibroblasts is primarily regulated by the TGF‐β pathway. The actions of TGF‐β are mediated by a cell surface receptor complex, composed of type I and type II TGF‐β receptors, and downstream effector Smad proteins (Smad2, 3 and 4), which are transcription factors. Smad proteins regulate many genes that encode for components of the ECM, including collagens, laminins, fibronectin and proteoglycans.^5^, ^9^, ^29^ In aged human skin, several components of the TGF‐β pathway are reduced.^9^, ^30^ Impairment of the TGF‐β pathway like accounts, at least in part, for diminished ECM production that is observed in aged skin. In this study, we used latrunculin A (Lat‐A), a potent inhibitor of actin,^31^, ^32^ to investigate mechanisms that link assembly of actin microfilaments to production of type I collagen, in human skin fibroblasts. We find that interference with actin polymerization impairs the TGF‐β signalling pathway through specific down‐regulation of TGF‐β type II receptor (TβRII). This down‐regulation is mediated, in part, by increased microRNA 21 (miR‐21), which directly targets TβRII synthesis. These data provide novel insights into mechanisms, by which disassembly of the actin cytoskeleton may deleteriously alter fibroblast function leading to age‐associated skin atrophy.

## 2 MATERIALS AND METHODS

### 2.1 Materials

MEM α, GlutaMAX™, Foetal Bovine Serum (FBS), 0.25% trypsin‐EDTA solution and Penicillin‐Streptomycin were purchased from GIBCO/Thermo Fisher (Wayne, MI, USA). Collagen I (rat tail) was obtained from BD Biosciences (Palo Alto, CA, USA). TGF‐β1 was obtained from R&D Systems (Minneapolis, MN, USA). Latrunculin A was obtained from Enzo Life Sciences (Farmingdale, NY, USA). TβRII siRNA (AACGGTGCAGTCAAGTTTCCA) and miR‐21 mimic (TAGCTTATCAGACTGATGTTGA) were purchased from Qiagen (Chatsworth, CA, USA). miR‐21 inhibitor (CAACATCAGTCTGATAAGCT) was purchased from EXIQON (Woburn, MA, USA). All other reagents were purchased from Sigma Chemical Company (St. Louis, MO, USA).

### 2.2 Cell culture

Primary adult human dermal fibroblasts were prepared from full‐thickness punch biopsies (4 mm) obtained from sun‐protected buttock skin of healthy adult human volunteers (mean age 35 ± 5 years). Dermal fibroblasts were isolated from skin biopsies by digestion with bacterial collagenase (Worthington Biochemical Corporation, Lakewood, NJ, USA). Early passage (less than 9 passages) dermal fibroblasts were cultured in MEM α, GlutaMAX™ with 10% Foetal Bovine Serum (FBS) at incubator with 37°C, 5% CO_2._ Three‐dimensional (3D*)* collagen gels were made following a previous publication with minor modification.^17^ Briefly, neutralized rat tail type I collagen was mixed in cocktail (DMEM, NaHCO3 [44 mmol/L], L‐glutamine [4 mmol/L], Folic Acid [9 mmol/L], and neutralized with 1N NaOH to pH 7.2). Fibroblasts (0.5 x 10^6^) were suspended in 2 mL collagen and medium cocktail solution then plated in 35 mm bacterial culture dishes. After collagen polymerization, collagen gels were incubated with 2 mL media (DMEM, 10% FBS) at 37°C, 5% CO_2_. For latrunculin A (Lat‐A) experiment, cells were treated with Lat‐A (30 nmol/L) for 24 hours.

### 2.3 RNA isolation and quantitative real‐time RT‐PCR

Total RNA was extracted using Trizol reagent (Invitrogen). cDNA was prepared by reverse transcription of total RNA(100 ng) using a TaqMan Reverse Transcription Reagents (Applied Biosystems, Foster City, CA, USA). Real‐time PCR was performed on a 7300 real‐time PCR system (Applied Biosystems) using SYBR Green PCR Master Mix (Applied Biosystems). Type I procollagen, CCN2 and TβRII primers were purchased from Applied Biosystems. Target gene mRNA expression levels were normalized to the housekeeping gene 36B4 for quantification. miR‐21 levels were determined by real‐time PCR using TaqMan^®^ MicroRNA Assays Kit (Life Technology, NY, USA) following the manufacturer's instructions.

### 2.4 Western analysis

Western blots were performed as described previously.^33^ Briefly, whole cell proteins were prepared using whole cell lysis buffer followed by centrifugation. Equal amount of proteins (50 μg/lane) were analysed for each sample by resolving on 10% SDS‐PAGE. The SDS gels were transferred to PVDF membrane; the membranes were blocked with 5% milk‐TBST for 1 hour at room temperature, followed by incubation with primary antibodies for 1 hour at room temperature. Primary antibodies: type I collagen (Southern Biotech, Birmingham, AL, USA), CCN2 (SC‐14939, L20, Santa Cruz Biotechnology, Santa Cruz, CA, USA), TβRI (SC‐398, V‐22, Santa Cruz Biotechnology), TβRII (SC‐400, L‐21, Santa Cruz Biotechnology), and total and phospho‐Smad3 (Cell Signalling Technology, Danvers, MA, USA). The membranes were washed with TBST then incubated with corresponding secondary antibodies for 1 hour at room temperature. Followed by three times TBST washing, the blots were incubated in ECF (Vistra ECF Western Blotting System, Amersham Pharmacia Biotech, Piscataway, NJ, USA) following the manufacturer's instructions. The blots were scanned by STORM MolecularImager (Molecular Dynamics, Sunnyvale, CA, USA). The intensities of each band were quantified using ImageQuant (GE HealthCare, Piscataway, NJ) and normalized using β‐actin as a marker for equal protein loading.

### 2.5 ProteinSimple capillary electrophoresis immunoassay

In some experiments, protein levels were determined by ProteinSimple capillary electrophoresis immunoassay. ProteinSimple capillary electrophoresis immunoassay overcomes many of the technical drawbacks of Western analysis while providing much greater sensitivity, in the low nanogram range. ProteinSimple capillary electrophoresis immunoassay was performed according to the ProteinSimple manufacture's user manual. In brief, whole cell extract samples (800 ng/lane) were mixed with kit provided master mix. The mixture was then heated at 95°C for 5 minutes. The samples including primary antibodies, blocking reagent, HRP‐conjugated secondary antibodies, chemiluminescent substrate and separation and stacking matrices were also dispensed to designated wells plate according to the ProteinSimple manufacture's user manual. The electrophoresis, blocking, washing and immunodetection steps took place in the capillary system (ProteinSimple Wes, ProteinSimple, Santa Clare, CA, USA) and were fully automated with instrument default settings. Corresponding protein bands from digital images were identified based on the molecular weight. The intensities of each protein were analysed by quantification with Compass software (ProteinSimple) after normalization by and β‐actin (loading control).

### 2.6 Transient transfection, immunostaining, and phalloidin staining

Primary adult human dermal fibroblasts were transiently transfected with TβRII expression vector^34^ or siRNAs (TβRII siRNA, miR‐21 mimic and inhibitor) by electroporation using human dermal fibroblasts nucleofector kit (Amaxa Biosystems, Gaithersburg, MD, USA). Immunocytochemistry was performed as described previously.^9^ Briefly, cells were fixed in 4% PFA (paraformaldehyde) for 2 hours at room temperature, followed by incubation with 0.5% Nonidet P‐40, blocking with 2% BSA (bovine serum albumin), washing with PBS five times/the cells were then incubated with type I procollagen (Santa Cruz Biotechnology, Santa Cruz, CA, USA), CCN2 (SC‐14939, L2, Santa Cruz Biotechnology), Phospho Smad3 (ab52903, abcam, Cambridge, MA,USA), TβRII (SC‐400, L‐21, Lot #: A1516, Santa Cruz Biotechnology) primary antibodies for 1 hour at room temperature, followed by incubation with Super Sensitive MultiLink (BioGenex, Fremont CA, USA) for 10 minutes and streptavidin‐conjugated AlexaFluor 594 or 488 (Invitrogen‐Molecular Probes, San Diego, CA) for 10 minutes. Mounting medium with DAPI was added to stain cell nuclei. Corresponding IgG isotype (negative control) show no specific staining (data not shown). Phalloidin was used to stain cells morphology.

### 2.7 Statistical analysis

Comparisons between samples were performed with the paired *t*‐test (two groups) or the repeated measures of ANOVA (more than two groups). Multiple pair‐wise comparisons among samples were made with the Tukey Studentized Range test. All *P* values are two‐tailed and considered significant when <.05.

## 3 RESULTS

### 3.1 Disassembly of actin cytoskeleton down‐regulates TGF‐β type II receptor

**Figure 1:**
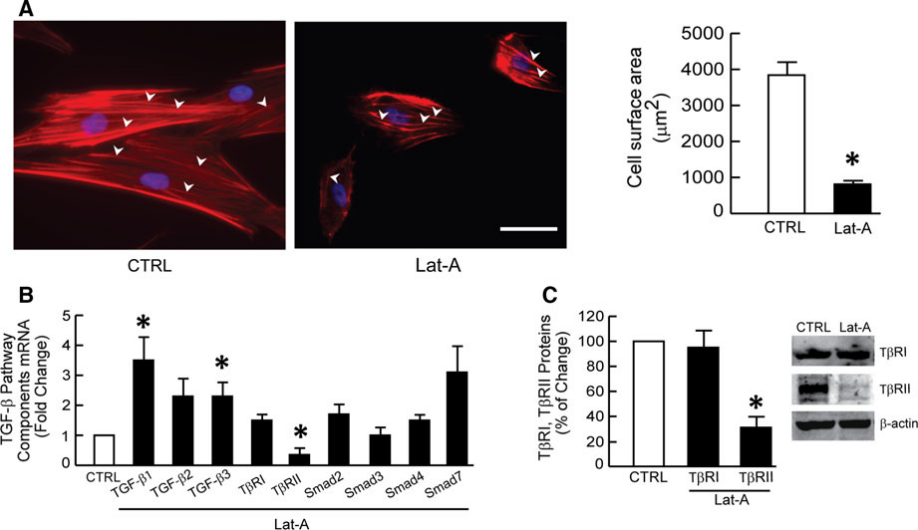
Actin cytoskeleton disassembly down‐regulates TGF‐β type II receptor. Dermal fibroblasts were treated with Lat‐A (30 nmol/L) or DMSO (CTRL) for 24 h. A, Dermal fibroblasts were stained with phalloidin and imaged by fluorescence microscopy. Red fluorescence delineates cell cytoplasm; blue fluorescence delineates nuclei. The relative cell surface areas were quantified by ImageJ software. Arrow heads indicate stretched actin fibres. Mean ± SEM, N = 6, *P < .05 vs CTRL. Scale bars = 100 μm. B, mRNA levels of TGF‐β pathway components were quantified by real‐time RT‐PCR and normalized to the housekeeping gene 36B4. Mean ± SEM, N = 3, *P < .05 vs CTRL. C, TβRI and TβRII protein levels were determined by Western blot and normalized to β‐actin (loading control). Band intensities were quantified by MolecularImager. Inset shows representative Western blot. Mean ± SEM, N = 3, *P < .05 vs CTRL

To explore the connection between the actin cytoskeleton and dermal fibroblast function, we disrupted the cytoskeleton with Lat‐A, which sequesters monomeric actin thereby causing rapid depolymerization of actin microfibrils.^35^ As expected, Lat‐A caused marked loss of actin stress fibres (Figure 1A, left panel) and reduced the average surface area of fibroblasts approximately 70% (Figure 1A, right panel).

We previously reported that the TGF‐β pathway is regulated by cell size and mechanical force regulate in dermal fibroblasts.^36^ When fibroblasts contract and generate less mechanical force, TGF‐β signalling is reduced.^27^ Therefore, we investigated whether disassembly of the actin cytoskeleton, which reduces fibroblast size and mechanical force, alters gene expression of TGF‐β pathway components. Cytoskeletal disassembly did not alter levels of TGF‐β ligand TGF‐β2, TGF‐β type I receptor (TβRI) or downstream effector Smads, Smad2, Smad3, Smad4 or Smad7 (Figure 1B). In contrast, cytoskeletal disassembly increased gene expression of TGF‐β ligands TGF‐β1 and TGF‐β3, and reduced expression of TGF‐β type II receptor (TβRII). This down‐regulation of TβRII mRNA was accompanied by significant reduction of TβRII protein (Figure 1C). TβRII protein was significantly decreased by 78%.

### 3.2 Actin cytoskeleton disassembly down‐regulates TGF‐β/Smad signalling and TGF‐β‐regulated type I procollagen and CCN2 expression

**Figure 2:**
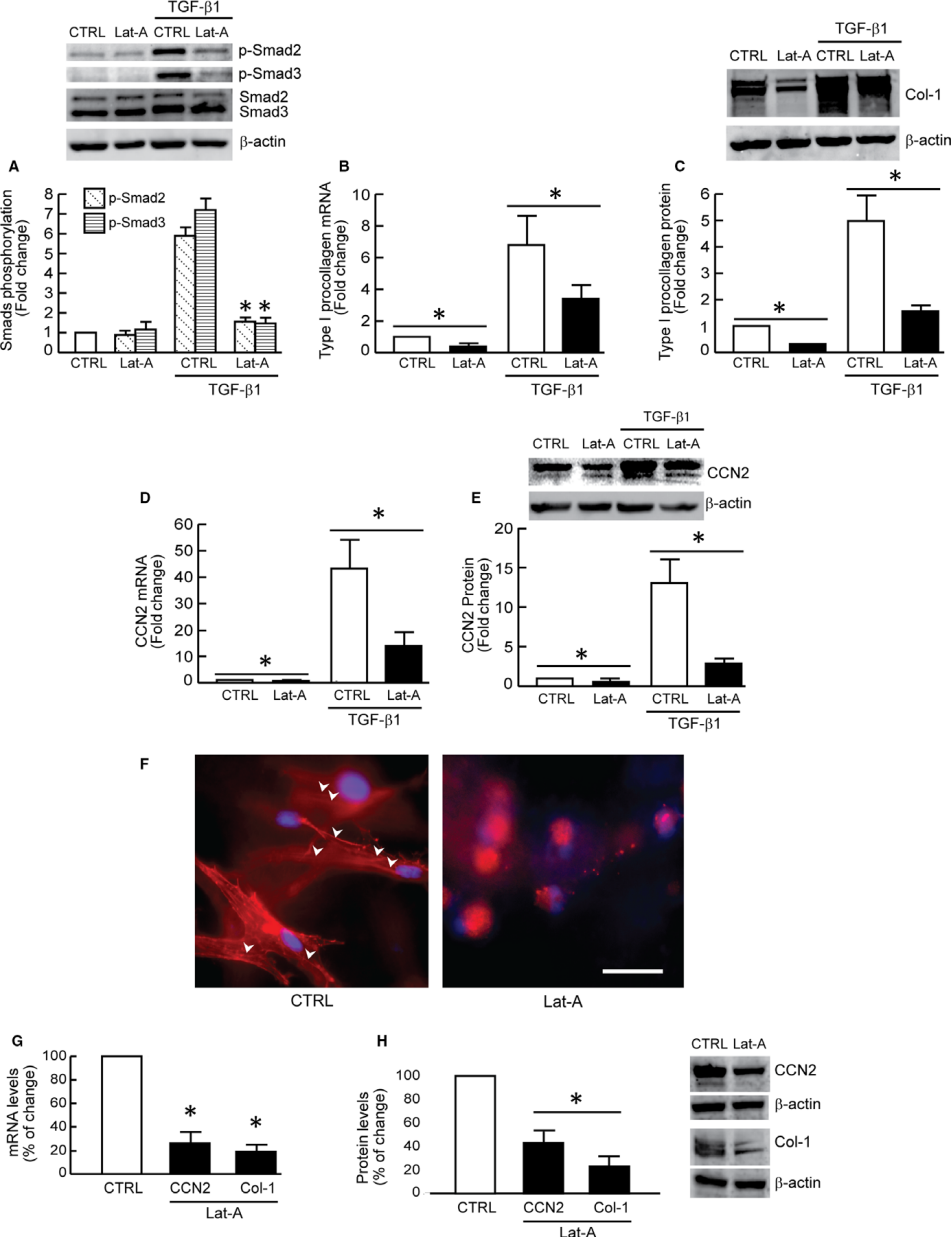
Actin cytoskeleton disassembly down‐regulates TGF‐β/Smad signalling and TGF‐β regulated type I procollagen and CCN2 expression. Dermal fibroblasts were treated with Lat‐A (30 nmol/L) or DMSO (control) for 24 h. A, Smad2/Smad3 phosphorylation. Cells were treated with TGF‐β1 (5 ng/mL) for 1 h. N = 3, *P < .05 vs CTRL with TGF‐β1. B, Type I procollagen mRNA levels. Mean ± SEM. N = 3, *P < .05. C, Type I procollagen protein levels. Mean ± SEM, N = 3, *P < .05. D, CCN2 mRNA levels. Mean ± SEM. N = 3, *P < .05. E, CCN2 protein levels. Mean ± SEM, N = 3, *P < .05. F, Cells were cultured in type I collagen lattices, stained with phalloidin and imaged by fluorescence microscopy. Red fluorescence delineates cell cytoplasm; blue fluorescence delineates nuclei. Arrow heads indicate stretched actin fibres. Representative image of three independent experiments. Scale bar = 100 μm. G, Type I procollagen and CCN2 mRNA levels. Mean ± SEM. N = 3, *P < .05 vs CTRL. H, Type I procollagen and CCN2 protein levels. Mean ± SEM. N = 3, *P < .05 vs CTRL. mRNA levels were quantified by real‐time RT‐PCR and normalized to the housekeeping gene 36B4. Protein levels were determined by Western blot analysis and normalized by β‐actin (loading control). Band intensities were quantified by MolecularImager. Inset shows representative Western blot

We next investigated the functional impact of actin cytoskeleton disassembly on TGF‐β signal transduction and target gene expression. Indeed, we found that TGF‐β‐induced phosphorylation of the downstream effectors Smad2 and Smad3 was significantly inhibited by disassembly of the actin cytoskeleton (Figure 2A). Phosphorylation of Smad2/3 is necessary for TGF‐β regulation of target genes. For example, Smad3 activation is necessary for TGF‐β induction of two genes that are critical for ECM function, type I procollagen and CCN2 (connective tissue growth factor).^9^ Actin cytoskeleton disassembly significantly reduced expression of both target genes. TGF‐β induction of type I procollagen mRNA (Figure 2B) and protein (Figure 2C) was reduced 58% and 76%, respectively. TGF‐β induction of CCN2 mRNA (Figure 2D) and protein (Figure 2E) was reduced 72% and 80%, respectively.

Dermal fibroblasts reside within a three‐dimensional (3D) collagen fibril‐rich ECM microenvironment in human skin. Accordingly, we investigated the impact of actin cytoskeleton disassembly on type I procollagen and CCN2 expression in fibroblasts cultured within 3D collagen lattices. In 3D collagen cultures, consistent with monolayer culture (Figure 1A), depolymerization of the actin cytoskeleton resulted in decreased fibroblast surface area (Figure 2F, right panel), and reduced expression of type I procollagen and CCN2 mRNA (Figure 2G) and protein (Figure 2H).

### 3.3 Actin cytoskeleton assembly stimulates TGF‐β signalling and type I procollagen expression

**Figure 3:**
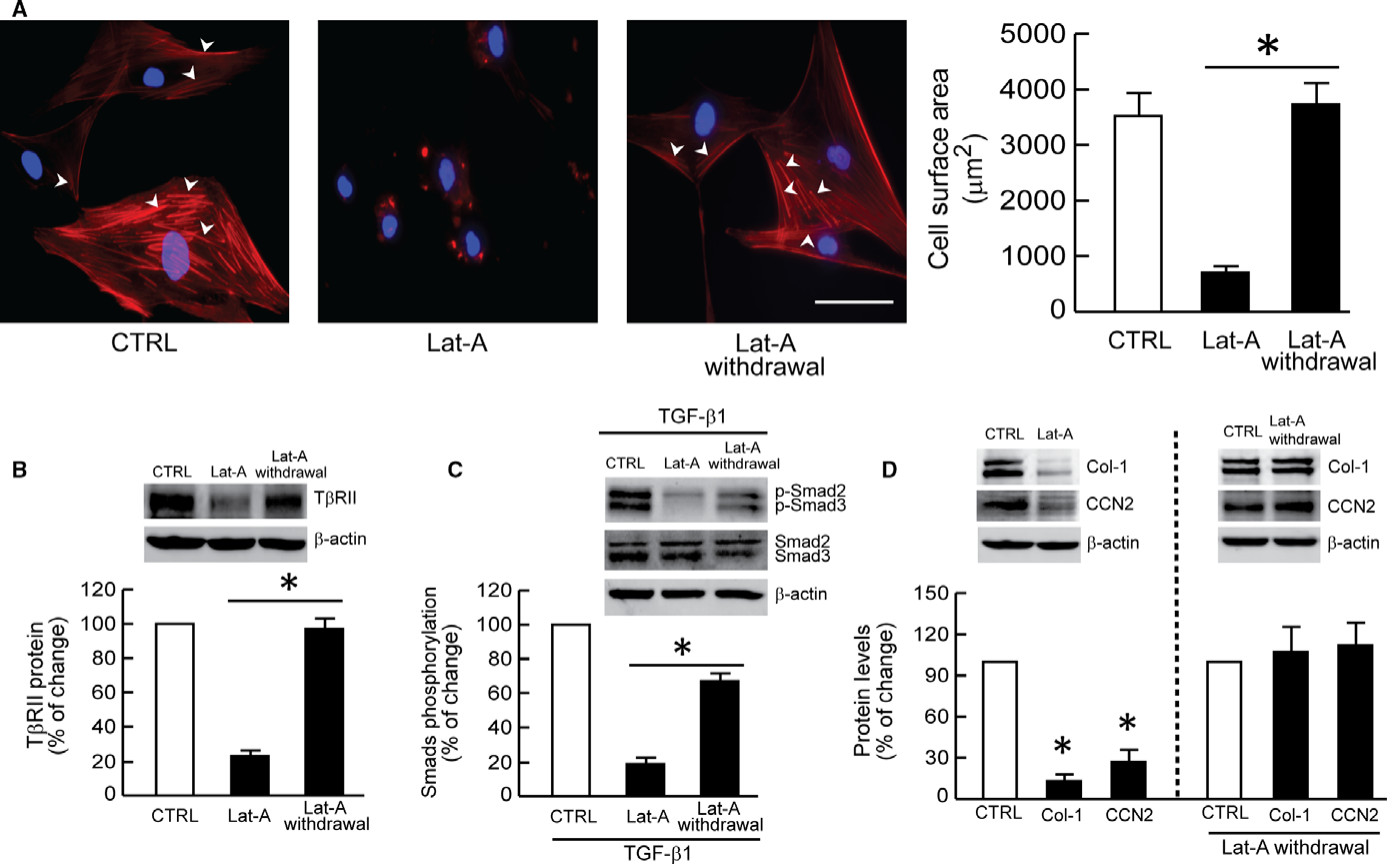
Actin cytoskeleton assembly stimulates TGF‐β signalling and type I procollagen expression. Dermal fibroblasts were treated with Lat‐A (30 nmol/L) or DMSO (control) for 24 h. Lat‐A‐containing media was withdrawn 24 h after its addition, followed by extensive washing with PBS and addition of fresh culture media. The cells were incubated for another 24 h after removal of Lat‐A. A, Cells were stained with phalloidin (red) to image the actin cytoskeleton and DAPI (blue) to image nuclei. The relative cell surface areas were quantified by ImageJ software. Arrow heads indicate stretched actin fibres. Mean ± SEM, N = 6, *P < .05. Scale bar = 100 μm. B, TβRII protein levels. Mean ± SEM, N = 4, *P < .05. C, Smad2/Smad3 phosphorylation. Cells were treated with TGF‐β1 (5 ng/mL) for 1 h. D, Type I procollagen (Col‐1) and CCN2 protein levels. Mean ± SEM, N = 3, *P < .05 vs CTRL. Protein levels were determined by Western blot analysis and normalized to β‐actin (loading control). Band intensities were quantified by MolecularImager. Inset shows representative Western blot

Inhibition of actin microfibril formation by Lat‐A has been shown to be rapidly reversible.^37^, ^38^ This reversibility provides a unique opportunity to control cytoskeletal assembly and determine its role in fibroblast functions. Therefore, we next assessed the impact of actin cytoskeleton assembly on TGF‐β signalling and gene regulation. For these studies, fibroblasts were first treated with Lat‐A or vehicle for 24 hours, and then Lat‐A‐containing media were replaced with normal media. Within 24 hours after removal of Lat‐A, distinct actin microfibrils were observed (Figure 3A). Importantly, TβRII protein levels were also restored to pre‐Lat‐A treatment levels (Figure 3B). This restoration of TβRII was associated with enhanced signal transduction, as demonstrated by increased TGF‐β‐induced Smad2/Smad3 phosphorylation (Figure 3C). In addition, consistent with up‐regulation of TGF‐β signalling, TGF‐β induction of both type I procollagen and CCN2 protein levels was significantly elevated (Figure 3D).

### 3.4 TβRII down‐regulation mediates impaired TGF‐β signalling and type I procollagen production by actin cytoskeleton disassembly

**Figure 4:**
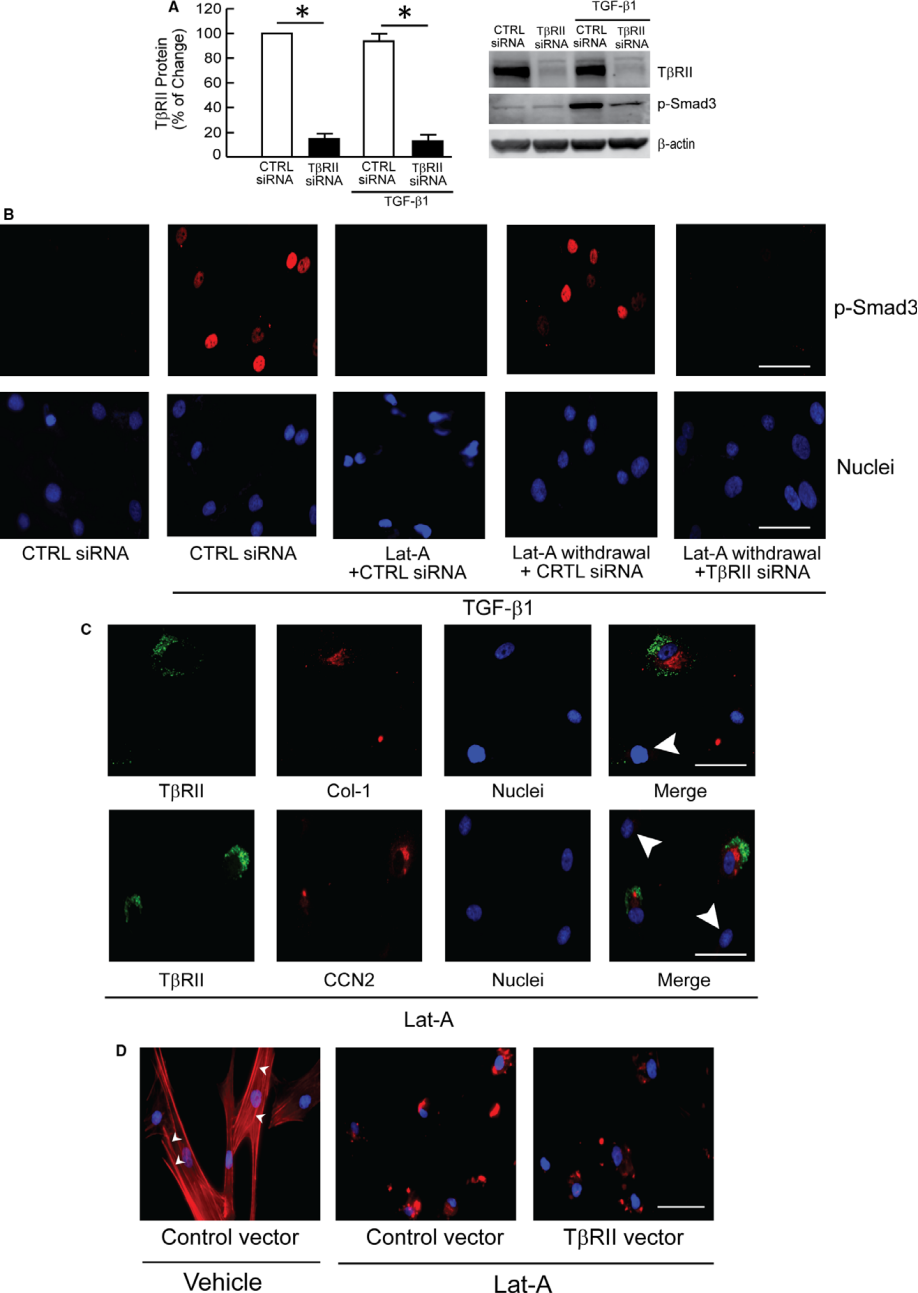
Down‐regulation of TβRII mediates impaired TGF‐β signalling and type I procollagen production by actin cytoskeleton disassembly. A, Knockdown of TβRII expression by TβRII siRNA. Cells were transfected with control siRNA or TβRII siRNA. Two days, cells were treated with vehicle or TGF‐β1 (5 ng/mL) for 1 h. TβRII and Smad3 phosphorylation (p‐Smad3) was determined by Western blot. Protein levels were normalized to β‐actin (loading control). Band intensities were quantified by MolecularImager. Inset shows representative Western blot. Mean ± SEM, N = 3, *P < .05 vs CTRL. B, Dermal fibroblasts were treated with Lat‐A (30 nmol/L) or DMSO (control, CTRL) for 24 h. Culture media were removed 24 h later, followed by extensive washing with PBS and replacement with fresh media alone (Lat‐A withdrawal) or containing Lat‐A. Cells were transfected with control siRNA or TβRII siRNA, as indicated. Twenty‐four hours later, cells were treated with vehicle or TGF‐β1 (5 ng/mL) for 1 h. Smad3 phosphorylation (p‐Smad3) was determined by immunostaining (red). Nuclei were stained with DAPI (blue). Scale bars = 100 μm. C, Fibroblasts were transfected with TβRII expression vector, and 48 h later treated with Lat‐A (30 nmol/L) for 24 h. Cells were stained for TβRII (green) and type I procollagen (Col‐1, red, top row), or CCN2 (red, lower row) by immunofluorescence. Nuclei were stained with DAPI (blue). Arrow heads indicate cells with reduced TβRII. Of note, these cells also displayed reduced expression of Col‐1 and CCN2. Scale bars = 100 μm. D, Cells were transfected with control vector or TβRII expression vector for 48 h, then treated with vehicle or Lat‐A (30 nmol/L) for 24 h. The actin cytoskeleton was stained with phalloidin (red) and imaged by fluorescence microscopy. Nuclei were stained with DAPI. Arrow heads indicate stretched actin fibres. All images are representative of three independent experiments. Scale bars = 100 μm

The above data indicate that the TGF‐β pathway is regulated by the state of actin cytoskeleton assembly and suggest that TβRII is a critical mediator of this regulation. To investigate this possibility, we determined the impact of loss or gain of TβRII. We confirmed that TβRII siRNA efficiently reduced the expression of TβRII and TGF‐β1 induced Smad3 phosphorylation (Figure 4A). As expected, removal of Lat‐A restored TGF‐β signalling (Figure 4, fourth panel from the left). In contrast, siRNA‐mediated knockdown of TβRII blocked restoration of TGF‐β signalling following removal Lat‐A (Figure 4B, last panel). We also found that prevention of TβRII down‐regulation, by transfection of TβRII expression vector, blocked down‐regulation of type I procollagen (Figure 4C, upper row) and CCN2 (Figure 4C, lower row) protein expression, following Lat‐A‐mediated actin cytoskeleton disassembly. In contrast, expression of TβRII did not prevent Lat‐A‐mediated actin cytoskeleton disassembly (Figure 4D). These data demonstrate that modulation of TβRII expression in response to the state of actin cytoskeleton assembly plays a key role in regulation of TGF‐β signalling and production of type I collagen and CCN2.

### 3.5 Reduction of fibroblast size *per se* does not down‐regulate TGF‐β signalling or TβRII

Integrins are cell surface receptors that bind to the ECM and link the ECM to the intracellular actin cytoskeleton. Integrins thereby play a critical role in normal cell attachment and spreading.^39^, ^40^ Disassembly of the actin cytoskeleton reduces cell spreading. We investigated whether reduced fibroblast spreading, under conditions of integrin‐independent cell attachment, exerts similar effects on TGF‐β signalling as reduced cell spreading due to cytoskeleton disassembly by Lat‐A treatment. For these studies, dermal fibroblasts were cultured on poly‐L‐lysine coated surfaces, which allow integrin‐independent attachment and spreading.^41^ Under these conditions, fibroblasts displayed limited spreading and reduced cell surface area (Figure S1A left panel), compared to cultures on uncoated, standard tissue culture plates (Figure S1B left panel). Interestingly, this reduced spreading did not alter TGF‐β‐induced Smad3 phosphorylation (Figures S1A,B) or TβRII expression (Figure S1C), compared to fully spread fibroblasts.

To further explore the relationship between reduced fibroblast spreading and TGF‐β signalling, we examined cells at early times after attachment to standard tissue culture plates, prior to full spreading. Although fibroblasts displayed limited spreading at 30 and 60 minutes after attachment, compared to 24 hours after attachment, TGF‐β‐induced Smad3 phosphorylation was similar at all time‐points.

### 3.6 Actin cytoskeleton disassembly induces miR‐21, which reduces TβRII expression

**Figure 5:**
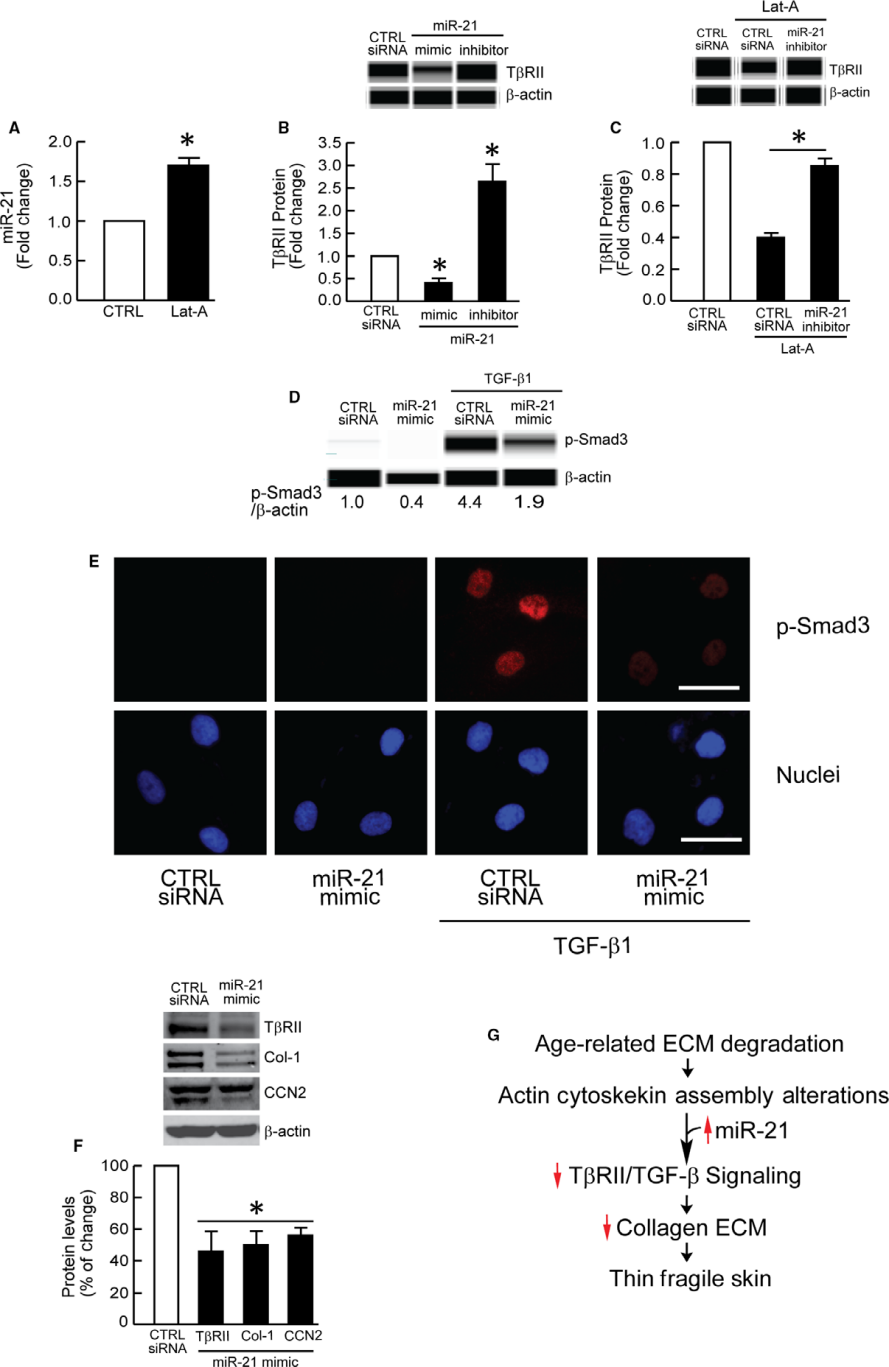
Actin cytoskeleton disassembly induces miR‐21, which reduces TβRII expression. A, miR‐21 expression levels. Dermal fibroblasts were treated with Lat‐A (30 nmol/L) or DMSO (CTRL) for 24 h. miR‐21 levels were determined by real‐time PCR and normalized to RNU6B, endogenous reference siRNA. Mean ± SEM, N = 3, *P < .05 vs CTRL. B, TβRII protein levels. Cells were transfected with control siRNA, miR‐21 mimic or miR‐21 inhibitor for 48 h. Protein levels were determined by capillary electrophoresis immunoassay. Mean ± SEM, N = 3, *P < .05 vs CTRL siRNA. C, TβRII protein levels. Cells were transfected with control siRNA or miR‐21 inhibitor for 48 h, then treated with Lat‐A (30 nmol/L) or DMSO (control) for 24 h. Protein levels were determined by capillary electrophoresis immunoassay. Mean ± SEM, N = 3, *P < .05. D, miR‐21 mimic inhibits Smad3 phosphorylation. Cells were transfected with control siRNA or miR‐21 mimic for 2 d. Cells were treated with vehicle or TGF‐β1 (5 ng/mL) for 1 h. Smad3 phosphorylation was determined by capillary electrophoresis immunoassay. E, miR‐21 mimic inhibits Smad3 phosphorylation. Cells were transfected with control siRNA or miR‐21 mimic for 2 d. Cells were treated with vehicle or TGF‐β1 (5 ng/mL) for 1 h. Smad3 phosphorylation (p‐Smad3) was determined by immunostaining (red). Nuclei were stained with DAPI (blue). Images are representative of three independent experiments. Scale bars = 50 μm. F, miR‐21 mimic inhibits type I collagen and CCN2 expression. Cells were transfected with control siRNA or miR‐21 mimic for 2 d. Type I procollagen and CCN2 protein levels were determined by Western blot and normalized to β‐actin (loading control). Band intensities were quantified by MolecularImager. Inset shows representative Western blot. Mean ± SEM, N = 3, *P < .05 vs CTRL siRNA. Protein levels (B‐D) were determined by capillary electrophoresis immunoassay and normalized to β‐actin (loading control). Band intensities were quantified by Compass software. Inset shows representative digital images. G, Diagram illustrating proposed mechanism of age‐related skin thinning and fragility. Collagen fibril fragmentation reduced fibroblast attachment, and actin cytoskeleton disassembly act in concert to up‐regulate miR‐21, which in turn impairs TGF‐β signalling via specific down‐regulation of TβRII. Impaired TGF‐β signalling reduces production of collagen and other ECM proteins, which eventuates in loss of dermal mass, giving rise to thin, fragile skin, a prominent feature of aged human skin

Finally**,** we explored the potential mechanism by which actin cytoskeleton disassembly reduces TβRII expression. Several recent reports indicate that TβRII expression is largely regulated by microRNA 21 (miR‐21) in both stromal (adipose tissue‐derived mesenchymal stem cells) and epithelial cells (colon cancer cell lines, HCT‐116 and HT‐29).^42^, ^43^ miR‐21 regulates TβRII expression through direct interaction with the 3′ non‐translated region in the TβRII transcript. Interestingly, we observed that actin cytoskeleton disassembly leads to significant induction of miR‐21 levels (Figure 5A). Furthermore, we found that miR‐21 mimic significantly down‐regulated TβRII protein level, whereas miR‐21 inhibitor significantly increased TβRII protein level in human dermal fibroblasts (Figure 5B). Importantly, blocking miR‐21 induction by miR‐21 inhibitor prevented suppression of TβRII by actin cytoskeleton disassembly (Figure 5C). We also confirmed that miR‐21 mimic inhibited Smad3 phosphorylation (Figure 5D,E) and reduced the expression of TβRII, and TGF‐β target genes, type I collagen and CCN2 (Figure 5F).

## 4 DISCUSSION

Fibroblasts in young skin attach to dense, intact collagen fibrils, which compose the bulk of skin dermal ECM connective tissue. This attachment promotes assembly of the actin cytoskeleton and generation of mechanical force. During ageing, dermal collagen fibrils become fragmented and disorganized.^17^, ^18^, ^20^ This degeneration of the dermal ECM impairs fibroblast attachment and consequent assembly of the intracellular actin cytoskeleton. Decline of collagen production by fibroblasts is a prominent feature of skin ageing.^5^, ^6^, ^9^, ^44^ Emerging evidence indicates that cell‐substrate attachment and assembly of the actin cytoskeleton, play critical roles in diverse cellular biological processes, such as proliferation, differentiation, signal transduction and gene expression.^22^, ^23^ Here, we explored the impact of disassembly of the actin cytoskeleton on the regulation of collagen production by dermal fibroblasts. Our results indicate that disassembly of actin cytoskeleton leads to down‐regulation of collagen production via impairment of TGF‐β/Smad signalling. These data suggest that reduced assembly of the actin cytoskeleton in aged skin, due to fragmentation of collagen fibrils in the dermal ECM, may contribute to age‐related collagen loss, in human skin.

The TGF‐β/Smad signalling pathway is a major regulator of ECM production.^9^, ^31^, ^45^, ^46^ TGF‐β initiates its biological actions through interactions with the TGF‐β receptor complex.^47^ TGF‐β binding to TβRII is required to initiate the TGF‐β signalling pathway. Binding of TGF‐β to TβRII triggers its association with TβRI, which phosphorylates and activates downstream Smad effectors. We found that actin cytoskeleton disassembly down‐regulated TβRII, without affecting TβRI expression. Three distinct isoforms of TGF‐β (β1, β2 and β3) have been identified in mammalian cells.^48^ Although they are structurally homologous, TGF‐β1 and TGF‐β3 bind to TβRII with higher affinity than TGF‐β2 in most cells.^49^, ^50^ Cytoskeleton disassembly modestly induced TGF‐β1 and TGF‐β3. This induction may reflect a compensatory response to down‐regulation of TβRII. In skin fibroblasts, reduction of TβRII, without reduction of TβRI, results in loss of cellular binding of TGF‐β and impairment of downstream Smad signalling.^51^ Thus, cytoskeleton disassembly impairs the first step of TGF‐β signalling by substantially reducing TβRII expression.

The actin cytoskeleton plays a critical role in the maintenance of the structural integrity of the cell and diverse dynamic cellular functions. As such, disassembly of actin filaments affects many cellular processes. Our data identify TβRII expression as the major mechanism, by which actin cytoskeleton assembly regulates collagen production. Actin cytoskeleton assembly is known to regulate other pathways, including Hippo signalling^52^ and MRT/SRF dependent gene expression.^26^

Integrins function as primary cell surface receptors for attachment of cells to the ECM. As such, integrins serve as physical links between the ECM and the intracellular actin cytoskeleton. Together, the actin cytoskeleton and integrins generate mechanical force. We found that limited fibroblast spreading, due to either integrin‐independent cell attachment, or at early times after integrin‐mediated attachment, does not reduce TβRII or impair TGF‐β signalling (Figures S1 and S2). These data support the conclusion that actin cytoskeletal disassembly, rather than reduced fibroblast spreading *per se*, down‐regulates TβRII, leading to reduced collagen production.

Interestingly, miR‐21, a potent inhibitor of TβRII expression,^42^, ^43^ is induced by actin cytoskeleton disassembly and mediates TβRII down‐regulation. These data suggest that expression of miR‐21 may be regulated, in part, by cellular mechanical sensing mechanisms. The transcription factor activator protein 1 (AP‐1) has been reported to directly regulate expression of miR‐21 through several response elements in the miR‐21 promoter.^53^ We have previously reported that reduced mechanical force elevates AP‐1 activity in human dermal fibroblasts,^17^ suggesting that miR‐21 elevation in response to actin cytoskeleton disassembly may result from activation of AP‐1. Additionally, we reported that reduced mechanical force increases reactive oxygen species (ROS), which induce AP‐1,^54^ in human dermal fibroblasts.^17^, ^56^ Interestingly, elevated ROS specifically down‐regulates TβRII, without affecting TβRI.^34^ These data support the possibility that actin cytoskeleton disassembly may up‐regulate miR‐21 through ROS‐mediated AP‐1 activation, leading to down‐regulation of TβRII. Obviously, additional studies are needed to address the detailed molecular mechanism(s) of actin cytoskeleton‐specific down‐regulation of TβRII expression.

We and others have previously shown that many TGF‐β/Smad‐regulated ECM proteins are down‐regulated in aged human skin.^9^, ^44^ Importantly, we observed down‐regulation TβRII in naturally aged,^30^ photoaged,^30^ and acute ultraviolet‐irradiated human skin in vivo,^51^ with no change of TβRI expression. These data indicate that TβRII and TβRI are differentially regulated and that TβRII specific down‐regulation may play a key role in impaired TGF‐β/Smad signalling and reduced ECM synthesis that are observed in aged human skin.

Recently, we reported that enhancing mechanical support within the dermis by direct injection of dermal filler (cross‐linked hyaluronic acid) into aged human skin in vivo increases TβRII levels, and stimulates collagen and CCN2 expression.^56^, ^57^ These data indicate that dermal fibroblasts in aged human skin retain their capacity to up‐regulate the TGF‐β pathway and produce collagen. Thus, loss of structural integrity of the dermal ECM, with concomitant reduced attachment and actin cytoskeleton disassembly, may be drive the decline of collagen production by fibroblasts in aged human skin.

In summary, we report that disassembly of actin cytoskeleton leads to down‐regulation of type I collagen via impairment of TGF‐β/Smad signalling. Actin cytoskeleton disassembly up‐regulates miR‐21, which in turn impairs TGF‐β signalling via specific down‐regulation of TβRII. These findings provide mechanistic insight regarding reduced expression of TβRII and type I collagen, which are observed in aged human skin (Figure 5G).

## AUTHOR CONTRIBUTION

ZQ performed the experiments and analysed the data; TQ and GJF designed the experiments and wrote the manuscript; GJF and JJV discussed the analyses, interpreted and edited the manuscript.

## DISCLOSURE

The authors declare no competing interests that might be perceived to influence the results and discussion reported in this paper.
