# Supplementary material for: Actin cytoskeleton assembly regulates collagen production via TGF‐β type II receptor in human skin fibroblasts
Source: J Cell Mol Med. 2018 Jun 11;22(9):4085–96. doi: 10.1111/jcmm.13685 (PMC6111811; doi:10.1111/jcmm.13685)
Supplement: Supplementary file 1 [file JCMM-22-4085-s001.pdf]

Supplemental Figure 1

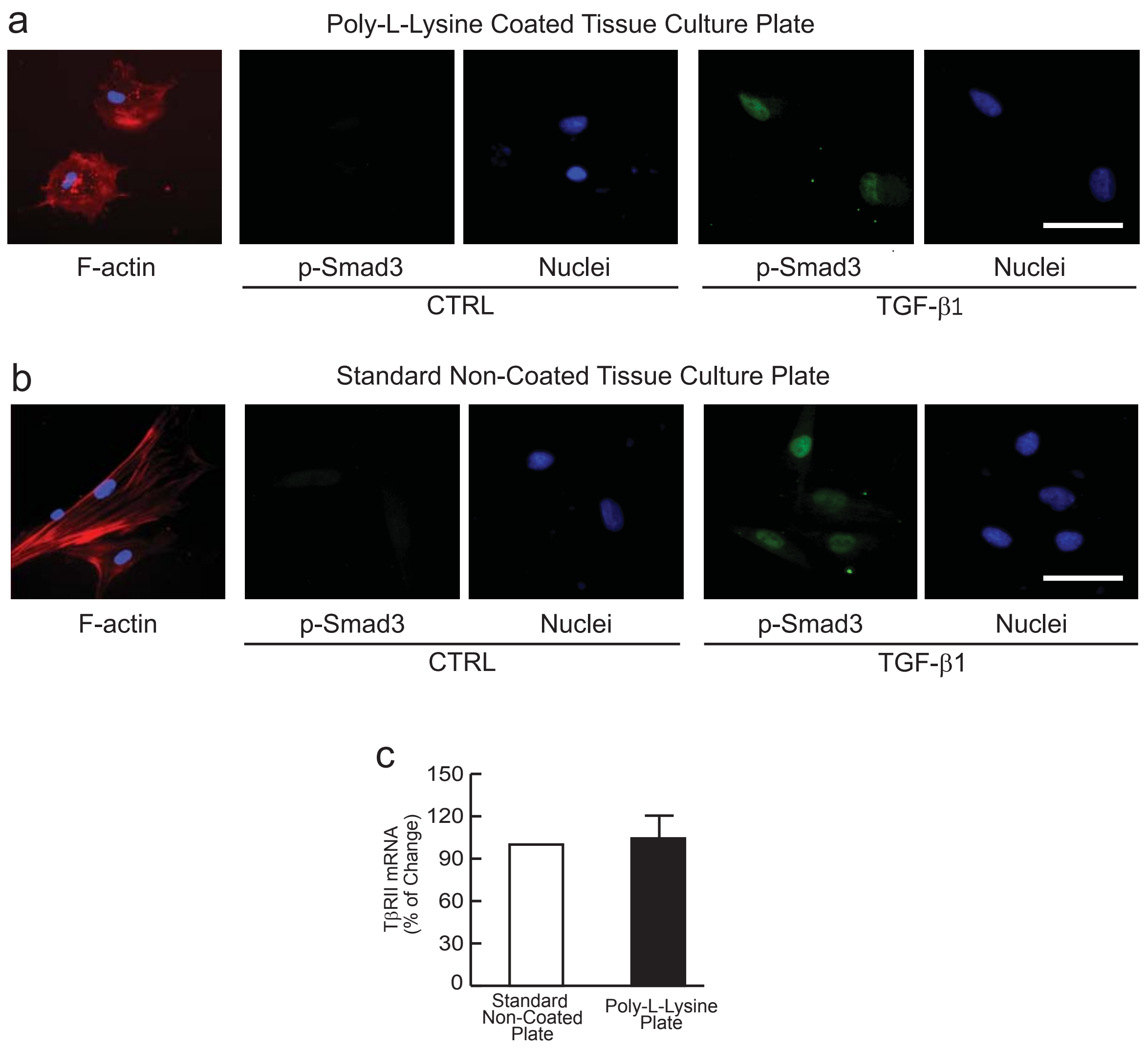

Supplemental Figure 1. Reduced fibroblast spreading due to attachment on poly-L-Lysine coated substrate does not alter TGF-β signaling. Dermal fibroblasts were cultured on (**a**) poly-L-lysine (PLL) coated plates (BioCoat™ Poly-L-Lysine Plates, Corning, NY, USA) or (**b**) standard uncoated tissue culture plates for 24 hours. Cells were stained with phalloidin (red) to image the actin cytoskeleton and DAPI (blue) to image nuclei. Cells were treated with vehicle or TGF-β1 (5ng/ml) for one hour. Smad3 phosphorylation (p-Smad3) was determined by immunostaining (green). Nuclei were stained with DAPI (blue). Scale bars = 100 μm. (**c**) TβRII mRNA levels were determined by real-time RT-PCR and normalized to the housekeeping gene 36B4.
Mean ± SEM, N=3.

Supplemental Figure 2

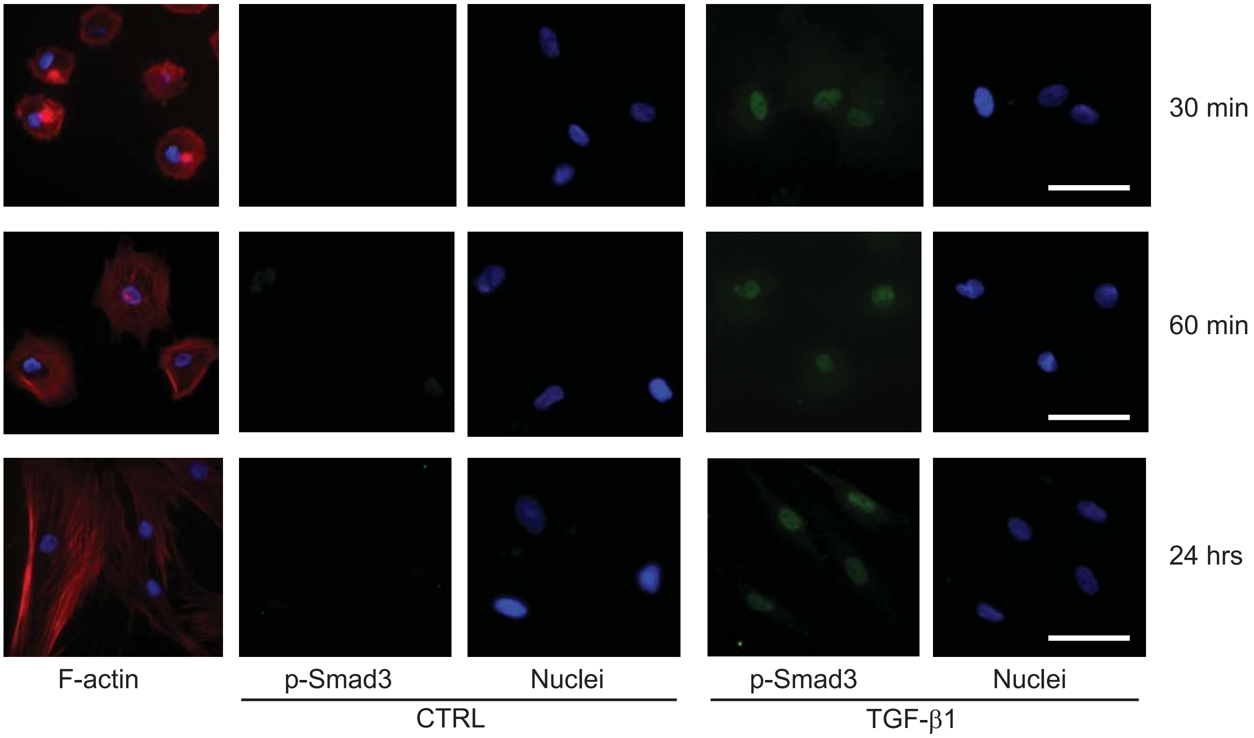

Supplemental Figure 2. Reduced cell spreading during early attachment does not alter TGF-β signaling. Dermal fibroblasts were cultured on standard tissue culture plates for 30 minutes (top panels), 60 minutes (middle panels), or 24 hours (bottom panels). Cells were stained with phalloidin (red) to image the actin cytoskeleton and DAPI (blue to image nuclei). Cells were treated with vehicle or TGF-β1 (5ng/ml) for one hour. Smad3 phosphorylation (p-Smad3) was determined by immunostaining (green). Scale bars = 100 μm. Images are representative of three independent experiments.
